# Kinetic and thermodynamic study in piezo degradation of methylene blue by SbSI/Sb_2_S_3_ nanocomposites stimulated by zirconium oxide balls

**DOI:** 10.1038/s41598-022-19552-3

**Published:** 2022-09-09

**Authors:** Karukh A. Babakr, Omid Amiri, L. Jay Guo, Mohammad Ali Rashi, Peshawa H. Mahmood

**Affiliations:** 1 Chemistry Department, College of Science, University of Raparin, Rania, Kurdistan Region Iraq; 2 Faculty of Chemistry, Razi University, Kermanshah, 6714414971 Iran; 3 Department of Electrical Engineering and Computer Science, University of Michigan, Ann Arbor, MI USA

**Keywords:** Environmental chemistry, Chemistry, Energy science and technology, Materials science, Nanoscience and technology

## Abstract

Mechanical energy harvesting by piezoelectric materials to drive catalysis reactions received extensive attention for environmental remediation. In this work, SbSI/Sb_2_S_3_ nanocomposites were synthesized as a catalyst. ZrO_2_ balls were used as an alternative mechanical force to ultrasonic for stimulating the piezocatalyst for the first time. The kinetics and thermodynamics of the piezo degradation of methylene blue (MB) were studied deeply. Besides the effect of the type of mechanical force, the number of ZrO_2_ balls, and temperature of the reaction on the degradation efficiency were studied. Here mechanical energy came from the collision of the ZrO_2_ balls with the catalyst particles. Using ZrO_2_ balls instead of ultrasonic vibration led to enhance degradation efficiency by 47% at 30 ± 5 °C. A kinetic study revealed that piezo degradation of methylene blue (MB) by SbSI/Sb_2_S_3_ catalyst followed pseudo-second-order kinetics. Based on thermodynamic results piezo degradation of MB was an exothermic reaction.

## Introduction

Developing alternative clean and sustainable energy sources has been drawing intense research interest in relieving environmental pollution and energy crises. Materials that can gather and convert solar or mechanical energy have been extensively researched in recent years as a new form of clean energy^1–8^. Piezo materials are considered an interesting class of material that can harvest and convert mechanical energy to electrical or chemical energy^9–12^. In this scenario, when the piezo material was forced under applied deformation caused by the mechanical force, the piezoelectric potential will shift the electronic energy levels of unoccupied or occupied states within the materials. In other words, it lowers the conduction band (CB) of the piezo material to below the highest occupied molecular orbital (HOMO) of the molecule of piezo material. Therefore, the electrons could transfer from the HOMOs of molecules to the CB of piezo material^13–17^. In the past, piezo materials mainly were used as sensors, transducers, and electronic industries^18–20^. Recently a new application was found, referred to as piezocatalysis. Till now, antibacterial and water splitting have been achieved via piezocatalysis. More recently, piezocatalysis were applied as a treatment agent to break down water pollutants which is one of the most severe environmental issues for human beings because some of these pollutants are highly soluble and chemically stable. Various types of materials were developed for this purpose, such as the 1D and 2 D wurtzite ZnO and BaTiO_3_^21,22^. For example, in 2019, Qian et al. reported decomposing of ~ 94% of rhodamine B (RhB) dye by using barium titanate (BaTiO_3_, BTO)–polydimethylsiloxane composite^23^. Later in 2020, Raju et al. applied Polyvinylidene Fluoride/ZnSnO_3_ Nanocube/Co_3_O_4_ composite to treat RhB and methylene blue (MB)^24^. Xu and his coworker degraded over 97% of RhB using Bi_0.5_Na_0.5_TiO_3_@TiO_2_ Composite^25^.

Employing piezocatalysis for environmental remediation has advantages compared to the other methods such as photocatalysis^26^, adsorbent^27^, and the Fenton process^28^. For example, photocatalysis only works under light. Besides, wide bandgap semiconductors are needed to avoid recombination of carrier charges. Using a wide-bandgap catalyst requires high-energy photons, and the material is usually more costly^29–34^.

Recently several articles published the mechanism behind the degradation of pollutants by piezoelectric materials. For example, we applied PbTiO_3_ nanostructures to treat acid red 143 and acid violet in water. We studied the possible mechanism by using radical scavengers and suggested that free radicals are responsible for degradation of organic pollutants^35^. Later in 2021, Lin et al. reported use of BaTiO_3_ nanocubes as piezo catalysts to treat organic pollutants and suggested the same mechanism^36^. Although several groups tried to study the possible mechanism behind it, the kinetics of degradation of organic pollutants by piezoelectric material is rarely studied. For instance, Lei et al studied the kinetics behind piezocatalytic degradation of dichlorophenols using two-dimensional graphitic carbon nitride. They reported that the degradation of dichlorophenols is pseudo-first-order kinetics^37^. However, the thermodynamics, effect of temperature, and amount of applied force on the kinetic of piezocatalytic degradation of pollutant were not discussed yet. Besides previous reports used ultrasonic actuation as a source of mechanical force, while we used zirconia balls to provide mechanical forces. Here we prepared piezo catalyst by sonochemistry and hydrothermal method. Then we studied the related kinetics at varied temperatures of 293 K, 303 K, and 313 K, and under different mechanical forces. Finally, we studied the thermodynamics behind the degradation.

## Experimental

### Material and characterization

Antimony sulfate (Sb_2_(SO_4_)_3_), Iodine, and sulfur were used as precursor materials without any purification and processing. 1.5 mm Diameter Zirconia Oxide Ceramic balls with hardness of HRA 87-91 were used to stimulate the piezo catalyst. The crystal structure of the samples was investigated by an X-ray diffractometer (Philips X'pert Pro MPD, The Netherlands) with Ni-filtered Cu Kα radiation (λ = 1.54 Å). The sonochemical process was performed by an ultrasonic bath of a 20 kHz ultrasonic device with a maximum output power of 250 W. EDS (energy dispersion spectroscopy) analysis was performed using an X-Max Oxford, England. ƩIGMA/VP- ZEISS, Germany was used to recording SEM images. To take the SEM images all samples were coated with gold. TEM images were captured by using transmission electron microscopy (TEM, Zeiss).

### Synthesis of SbSI/Sb_2_S_3_ nanocomposites by sonication method

To synthesize SbSI/Sb_2_S_3_ nanocomposites by sonochemistry method first 2 g of Sb_2_(SO_4_)_3_, 0.95 g of Iodine, and 0.24 g of sulfur weighted. Then, mixed those in 100 mL ethanol under stirring for 10 min. Afterward, the above solution was sonicated in an ultrasonic bath with 250 W in power for 2 h. Products were separated and washed several times, followed by drying overnight at 70 °C (sample S_1_). In the case of sample S_2_, the ultrasonic time was changed to 3 h.

### Preparation of SbSI/Sb_2_S_3_ nanocomposites by solvothermal method

The solvothermal method was used as an alternative method to prepare SbSI/Sb_2_S_3_ nanocomposites. In this case, 2 g of Sb_2_(SO_4_)_3_, 0.95 g of Iodine, and 0.24 g of sulfur were weighted and mixed in 40 mL of ethanol under stirring for 10 min. Afterward, the above mixture was transferred to stainless steel autoclave and heated at 180 °C for 6 h. finally, the product was separated and washed several times with water and ethanol and dried at 70 °C, This sample was labeled as S_3_. The next sample which was labeled as S_4_ was prepared with the same recipe and hydrothermal temperature of 245 °C. Samples S_5_ and S_6_ were prepared to study the effect of hydrothermal time on the morphology and purity of products. Hydrothermal time for samples S_5_ and S_6_ was 4 h and 8 h, respectively. The detail in the preparation of Samples S_1_–S_6_ could be found in Table S1 as supporting information.

### Evaluation of piezocatalytic activity of SbSI/Sb_2_S_3_ nanocomposites

The piezocatalytic activities of SbSI/Sb_2_S_3_ nanocomposites are evaluated by degrading Methylene blue (MB). 1 g L^−1^ of SbSI/Sb_2_S_3_ nanocomposites was added to the 12.5 mL of Methylene blue with different concentrations and was stirred in dark for 30 min to equilibrium adsorption–desorption of dye on the catalyst. In the case of using ultrasonic as mechanical force, the mixture of dye solution and catalyst was sonicated in dark at different times. After a certain time, the UV–Vis of samples was taken to monitor the degradation efficiency of MB. In the other reaction batches, zirconium oxide balls were used instead of ultrasonic to supply force to stimulate piezocatalyst. The numbers of zirconium oxide balls were optimized by studying the effect of different number of balls on the degradation of MB.

The effect of preparation condition of catalyst, time, temperature, type of mechanical force, amount of mechanical force was studied on the piezocatalytic activities of SbSI/Sb_2_S_3_ nanocomposites.

### Studying kinetic and thermodynamic of reaction

The study of reaction kinetics was performed by collecting the sample every 5 min while the temperature was controlled at the indicated temperatures. The experiments for piezo degradation isotherm evaluation were carried out in a batch system with the MB concentration range of 5 and 10 ppm at three different temperatures of 293 K, 303 K, and 313 K, during 90 min of reaction.

The pseudo-first-order rate constants and pseudo-second-order rate constants were determined by a non-linear curve fitting to the first-order and second-order reaction equation. Activation parameters were determined by fitting the data to the linearized form of the Eyring equation.

## Results and discussion

SbSI/Sb_2_S_3_ nanocomposites were prepared by modification of the sonication method according to the ref^38^. Besides, SbSI/Sb_2_S_3_ nanocomposites were prepared by the solvothermal method. The XRD and EDS of prepared samples were presented in Figure S1 and Figures S2–S7. XRD pattern of samples S_1_–S_6_ in Figure S1 showed that S_3_ had an amorphous structure, while other samples crystallized as orthorhombic SbSI and orthorhombic Sb_2_S_3_ closely matched well with the previous reports on the XRD pattern of SbSI nanostructures^39–42^. EDS results for the qualitative analysis of SbSI/Sb_2_S_3_ nanocomposites were presented in Figures S2–S7 and approved the presence of elements S, Sb, and I in prepared samples which could be assigned SbSI/Sb_2_S_3_ nanocomposites. In all spectra, the Au peak was observed due to the use of gold for surface conductivity for SEM analysis.

**Figure 1:**
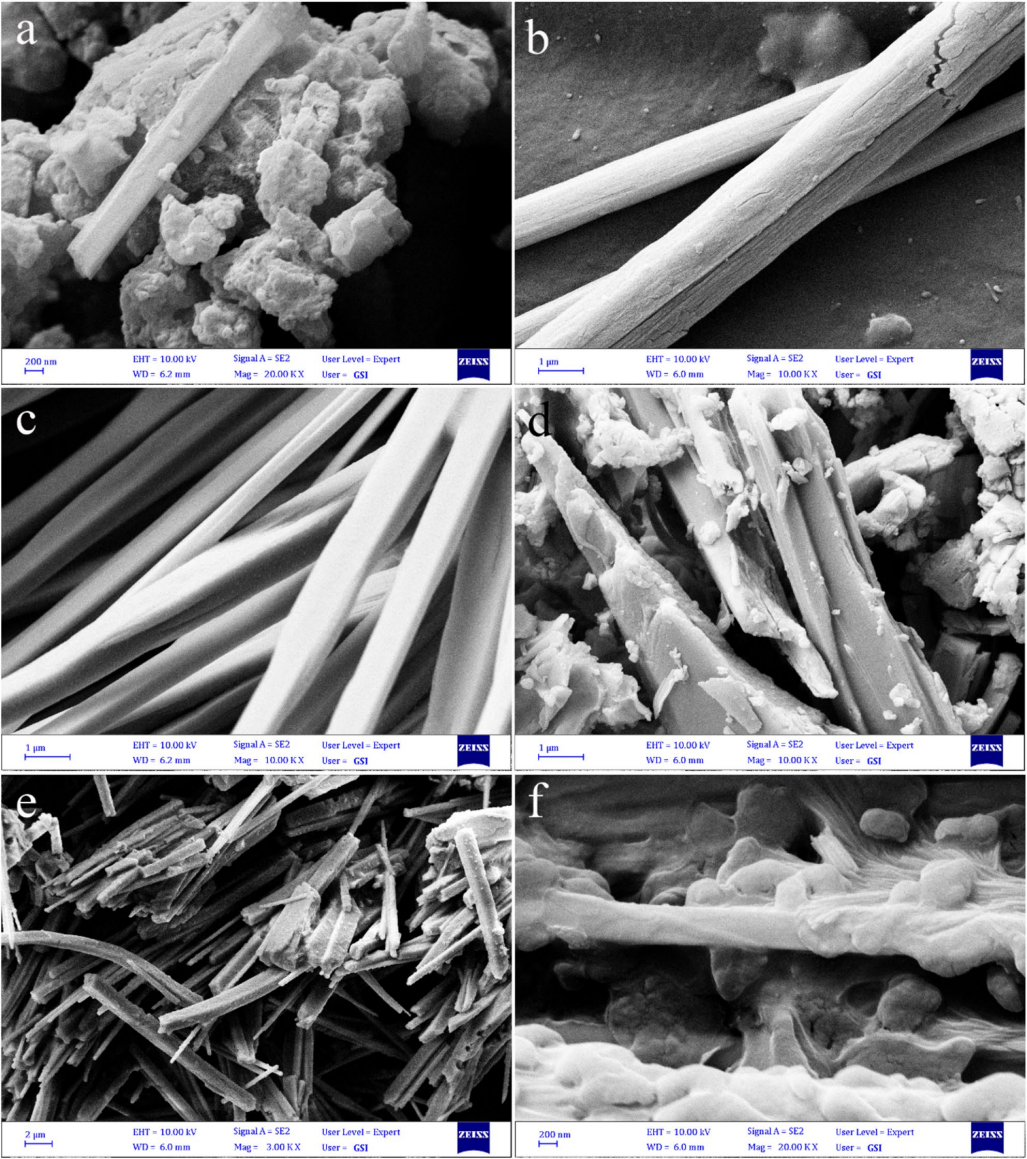
SEM images of piezo catalysts prepared (**a**) by sonochemistry for 2 h (S1), (**b**) sonochemistry for 3 h (S2), (**c**) by solvothermal method at 180 °C for 6 h (S3), (**d**) by solvothermal method at 245 °C for 6 h (S4), (**e**) by solvothermal method at 180 °C for 4 h (S5), and (**f**) by solvothermal method at 245 °C for 8 h (S6).

**Figure 2:**
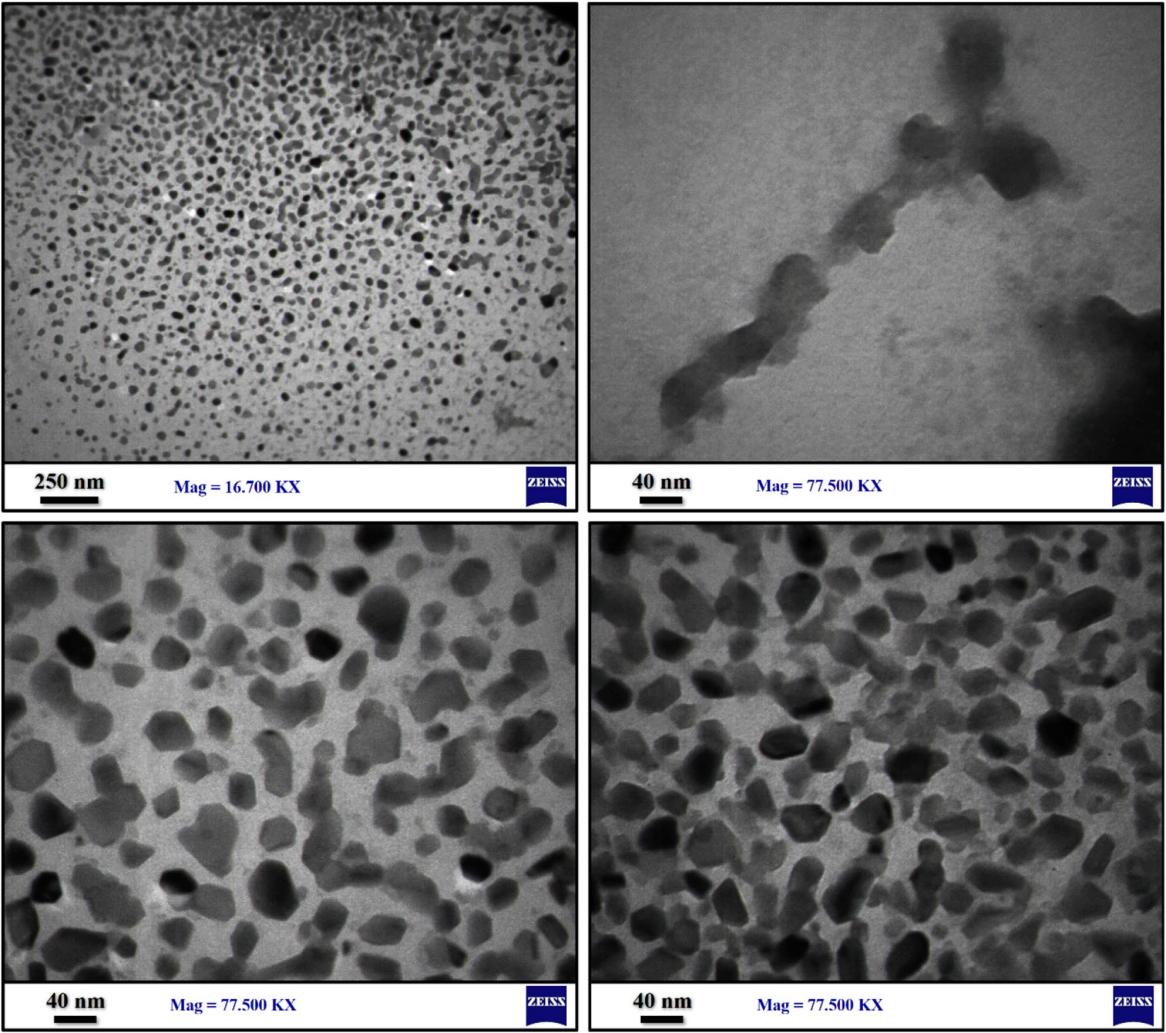
TEM images of sample prepared by solvothermal at 245 °C for 6 h (S4).

SEM images of samples S_1_–S_6_ was illustrated in Fig. 1a–f which showed that S_1_ mainly consists of aggregated nanoparticles (Fig. 1a). As depicted in Fig. 1b, uniform micro-size rods of SbSI/Sb_2_S_3_ nanocomposites were formed by increasing sonication time to 3 h. The morphology of the solvothermal synthesized SbSI/Sb_2_S_3_ nanocomposites at 180 °C for 6 h was depicted in Fig. 1c, where they exist in a regular rod shape with an average diameter of about 400 nm. Figure 1d displays the SEM image of the sample prepared by the solvothermal method at 245 °C where a mixture of more thick rods and nano-sized plates were formed. Changing the solvothermal time to 4 h at 180 °C led to the form of belt-like structures (Fig. 1e). The SEM image of sample S_6_ is shown in Fig. 1f, where very thick plate-like structures appeared. Due to the high reaction time belt-like structures stuck together and form thick plate-like structures. TEM images of sample S_4_ that was prepared by solvothermal at 245 °C were illustrated in Fig. 2. TEM images show that hexagonal shape nanostructures with an average size of 40–70 nm form rod-likes and plate-like structures.

### Piezocatalytical evaluation of SbSI/Sb_2_S_3_ nanocomposites

**Figure 3:**
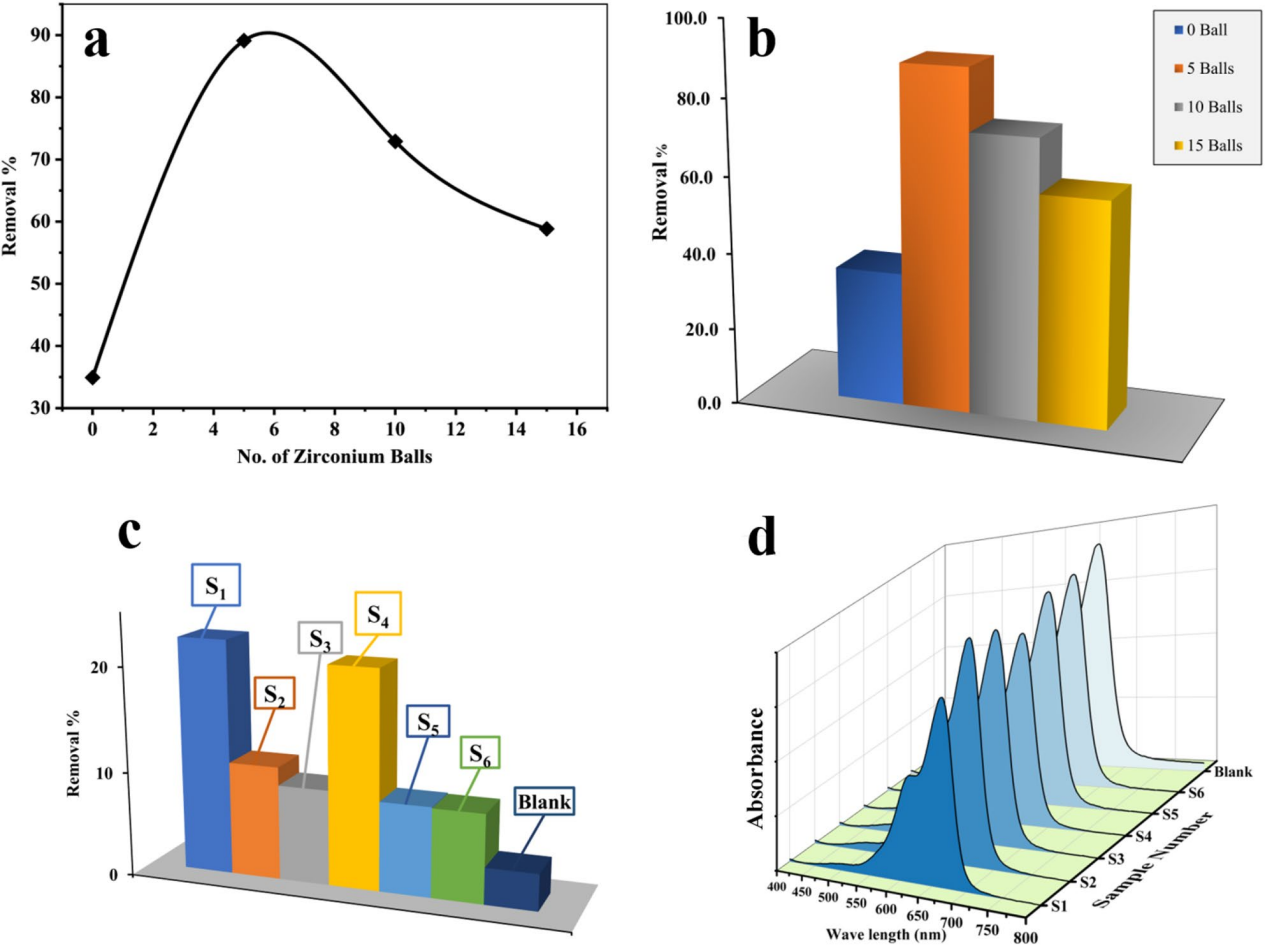
(**a**) Degradation efficiency of 10 ppm of MB in presence of sample S4 by using different ZrO_2_ balls as source of mechanical energy, (**b**) related spectrum of MB. (**c**) degradation efficiency of MB by using samples S-S6 with shaking speed of 250 RPM at 30 ± 2 °C for 1 h and related spectrum (**d**).

First, the effect of the number of ZrO_2_ balls added to the solution was studied by using 0, 5, 10, and 15 balls at 25 ± 5 °C. Results were summarized in Fig. 3a, b, it showed the degradation of 10 ppm MB was about 34% when there was no ball in the shaker (shaking RPM = 350). Degradation increased to 89.1% by adding 5 balls to the reactor. It happens because of increasing mechanical force produced by kinetic energy from the collision of the balls with the catalyst and the balls together. Increasing the number of balls to 10 led to a decrease in degradation efficiency to 72.9%. Finally, 15 balls were used in the reactor which degradation efficiency of 58.8% was achieved by using sample S_4_ as the catalyst. So 5 ZrO_2_ balls were used in the next studies, degradation in the presence of 5 balls was higher than 10 and 15 balls because 5 balls provided more kinetic energy (mechanical energy) to stimulate the catalyst. This can be seen in the detail about the momentum of balls in fluid and slow-motion videos of 5, 10, and 15 ZrO_2_ balls in water as a simulation of the degradation reactor of MB as presented in supporting information (Figure S8).

Another parameter that affects the degradation efficiency of MB by SbSI/Sb_2_S_3_ nanocomposites was the type and amount of mechanical force. Results for using a shaker in the presence of ZrO_2_ balls without a catalyst at 250 RPM, using a shaker in the presence of a catalyst at 250 RPM, using a shaker in presence of both the catalyst and ZrO_2_ balls at 150, 250, and 350 RPM, and using ultrasonic bath are presented in Figure S9 (supporting information). By applying ZrO_2_ balls without catalyst at 250 RPM and 30 ± 5 °C only 3.5% of MB was decomposed. Repeat the test in the presence of the catalyst and without balls at the same shaking speed and temperature leading to degrading 12.8% of MB. Adding catalyst and ZrO_2_ balls decompose 25.7% of MB at the same shaking speed and temperature. By increasing shaking speeds to 350 RPM in the presence of catalyst and balls, degradation efficiency increased to 67.2% at 30 ± 5 °C. However, 15.2% of MB was decomposed in the presence of catalyst and balls when the shaking speed decreased to 150 RPM. Finally, applying 250 W in power ultrasonic waves as mechanical force decomposed 45.7% of MB at 30 ± 5 °C.

The effect of different samples on the degradation of MB was investigated by using 5 balls. Differently prepared samples were used as piezo catalysts with less than 250 RPM at 30 ± 2 °C for 1 h and the results were depicted in Fig. 3c, d. Following degradation efficiencies were achieved for sample S_1_–S_6_, respectively: 22.4%, 9.1%, 10.8%, 20.8%, 8.6%, and 8.5%. The first sample prepared by ultrasonic for 2 h shows the highest degradation efficiency.

**Figure 4:**
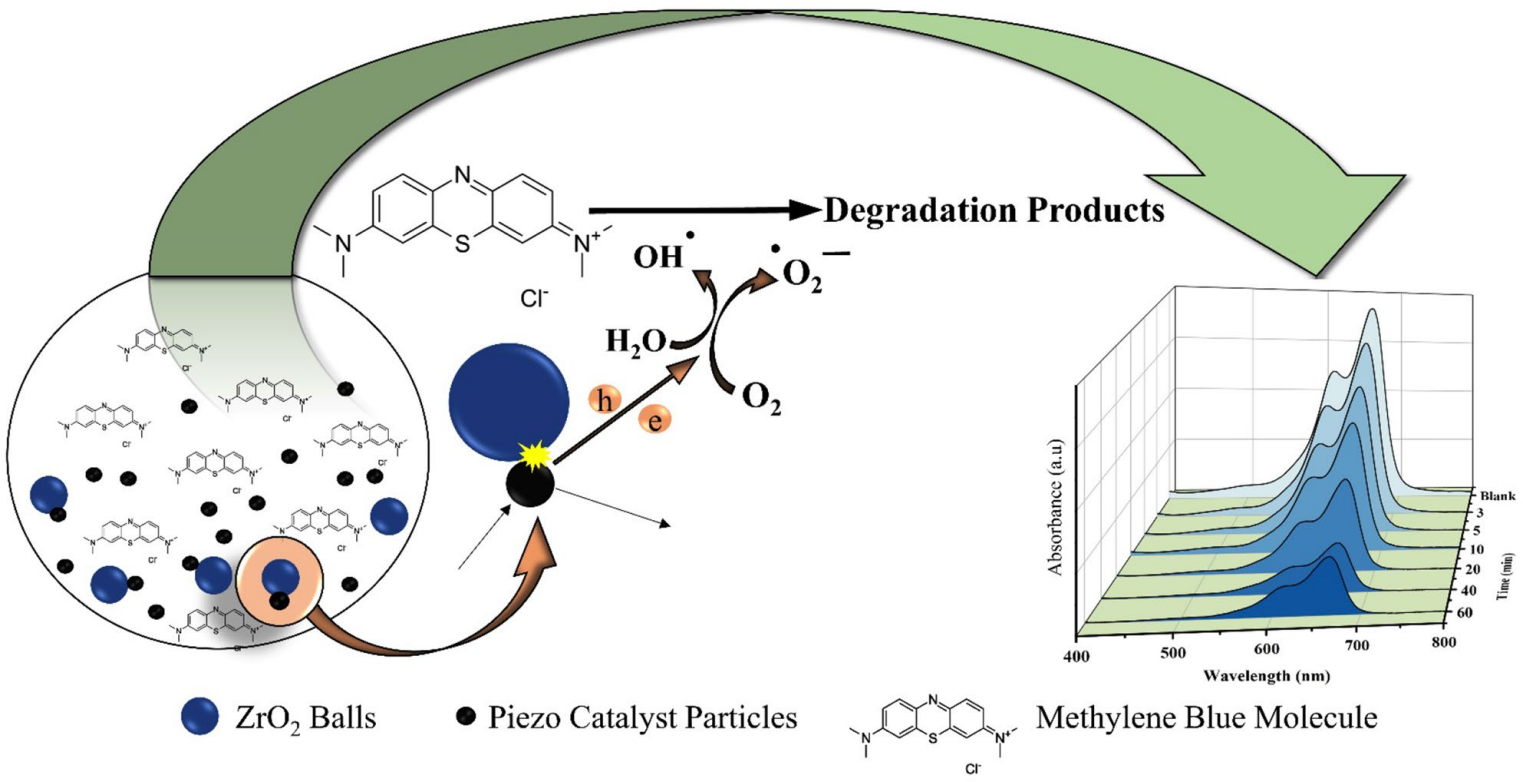
Schematically illustration of degradation of MB dye by SbSI/Sb_2_S_3_ nanocomposites in presence of ZrO_2_ balls.

The mechanism behind the degradation by ZrO_2_ balls and piezo catalyst is schematically illustrated in Fig. 4. When piezo catalyst particles collided with ZrO_2_ balls, electrons and holes were produced in the piezo catalyst. Generated electrons and holes reacted with oxygen and water molecules and produced oxidation radicals that could decompose dye pollutants.

**Figure 5:**
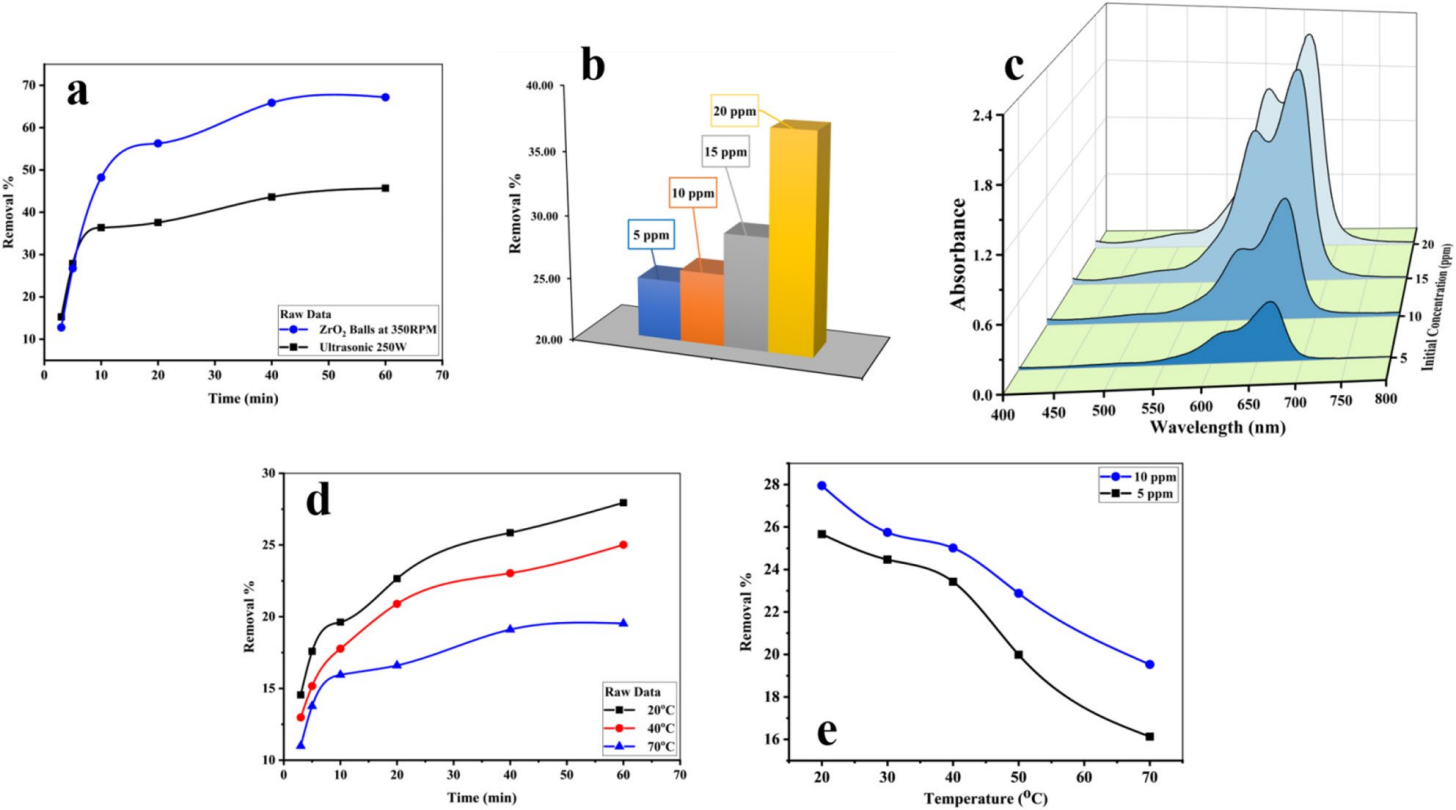
(**a**) Degradation MB by using S4 as a catalyst and ultrasonic bath with 250 W in power (red curve) and ZrO_2_ balls as vibration source (black curve), (**b**) removal percentages of different dye concentrations, and related spectrum (**c**), (**d**) degradation efficiency of 5 ppm and 10 ppm of MB in presence of sample S4 by using 5 ZrO_2_ balls. (**e**) Degradation percentages of MB degradation over time at 20 °C (brown curve), 40 °C (blue curve), and 70 °C (black curve).

Figure 5a, b illustrate the effect of the initial concentration of dye and shaking time on the degradation of MB. Figure 5a demonstrates the degradation of MB over time by using S_4_ as the catalyst at 30 ± 5 °C. 47.7% of MB was degraded during 1 h of sonication in an ultrasonic bath at 250 W. By repeating the test on the shaker at a shaking speed of 350 RPM, degradation efficiency increased to 67.2%. Sample S_4_ showed a lower degradation efficiency compared to the result presented in Fig. 3a because the experiment in Fig. 3a was done at a lower temperature, the effect of reaction temperature on degradation efficiency will be discussed later. The effect of the initial concentration of dye on the degradation efficiency of MB is presented in Fig. 5b, related spectrum is presented in Fig. 5c. Degradation efficiency for 5, 10, 15, and 20 ppm of MB was tested by using 5 balls at 250 RPM at 30 ± 0.5 °C. Based on these results, degradation efficiency was increased by increasing the initial dye concentration. In the case of 5 and 10 ppm, 24.7% and 25.7% of MB were degraded while 29% and 37% of MB were degraded in case of 15 and 20 ppm of pollutant.

Another parameter that significantly affects degradation efficiency in piezo degradation was temperature. To figure out this effect, piezo degradation was studied at 5 different temperatures including 20, 30, 40, 50, and 70 °C. As proven in Fig. 5d, degradation efficiency decreased over temperatures, and removal percentages of MB decreased from 25.7% and 27.9% for 5 and 10 ppm of MB at 20 °C with 250 RPM to 16.1% and 19.5% at 70 °C and 250 RPM. Figure 5e shows the degradation efficiency over time for 10 ppm MB at 20 °C, 40 °C, and 70 °C. Based on the results, degradation efficiency was decreased by more than 30% by increasing the temperature of shaking from 20 to 70 °C. This happens because we were close to the curie temperature by increasing the temperature and the catalyst will show a more symmetric structure^41,42^.

### Kinetic and thermodynamic study

The kinetics of degradation and characteristic constants of degradation by piezocatalyst can be studied through kinetic measurements using pseudo-first-order, pseudo-second-order, and intra-particle diffusion. The Lagergren pseudo-first-order model is given as follows^43,44^:

1 $$ \frac{dq}{{dt}} = {\text{k}}_{1} ({\text{q}}_{{\text{e}}} - {\text{q}}_{{\text{t}}} ) $$

where q_e_ and q_t_ (mg g^1^) are the amounts of the MB degraded at equilibrium and at time t (min). k_1_ (min^−1^) is the rate constant.

**Figure 6:**
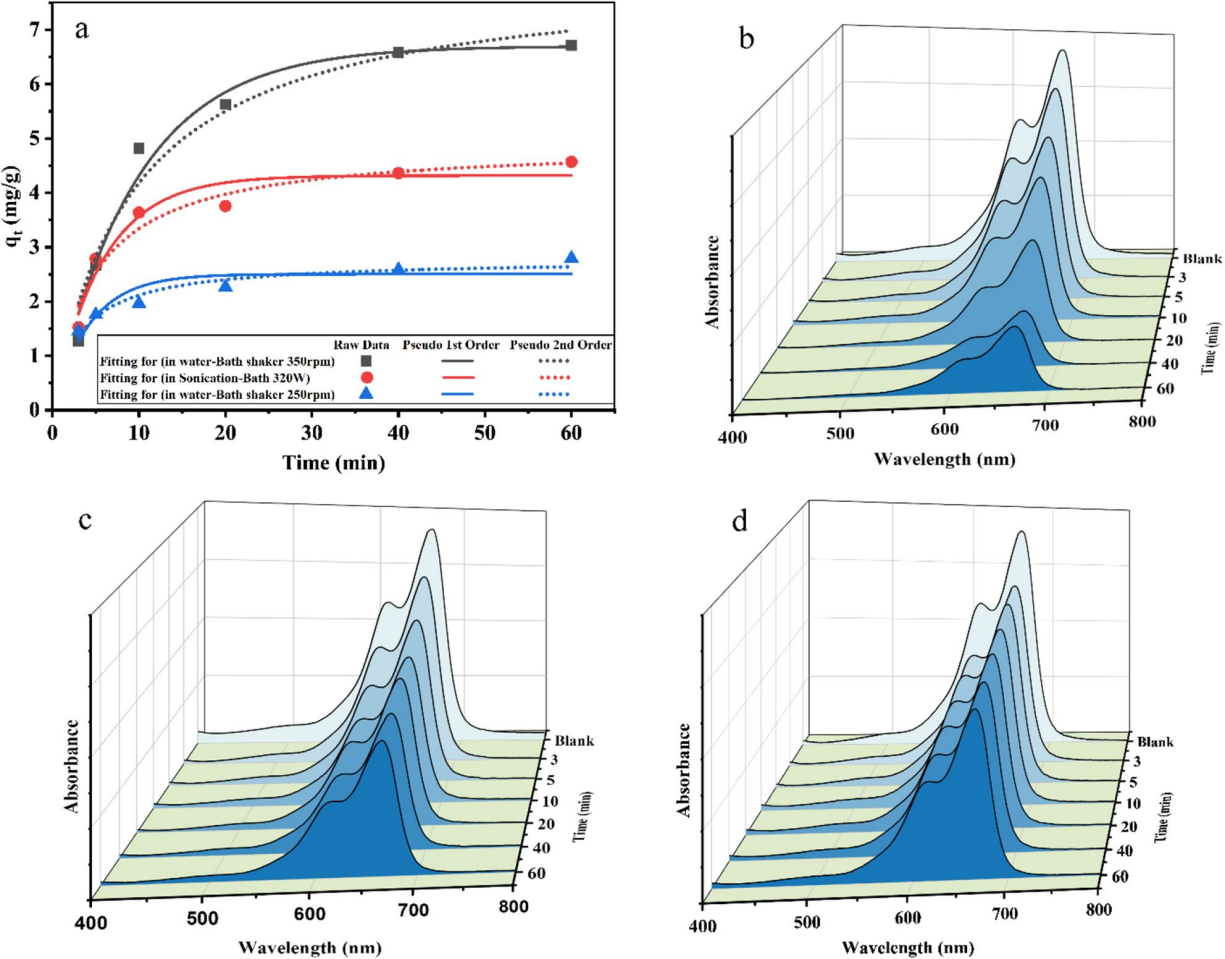
(**a**) Pseudo first-order and pseudo second-order kinetic models for degradation of 10 ppm MB in 250 W ultrasonic (red curve), reactor containing 5 ZrO_2_ balls shaking with 250 RPM (blue curve), and 350 RPM (black curve), (**b**) spectrum of MB over time in presence of S4 as catalyst and ZrO_2_ balls as source of mechanical force at 350 RPM. (**c**) spectrum of MB over time in presence of S4 as catalyst in ultrasonic bath with 250 W in power, (**d**) spectrum of MB over time in presence of S4 as catalyst and ZrO_2_ balls as source of mechanical force at 250 RPM.

**Figure 7:**
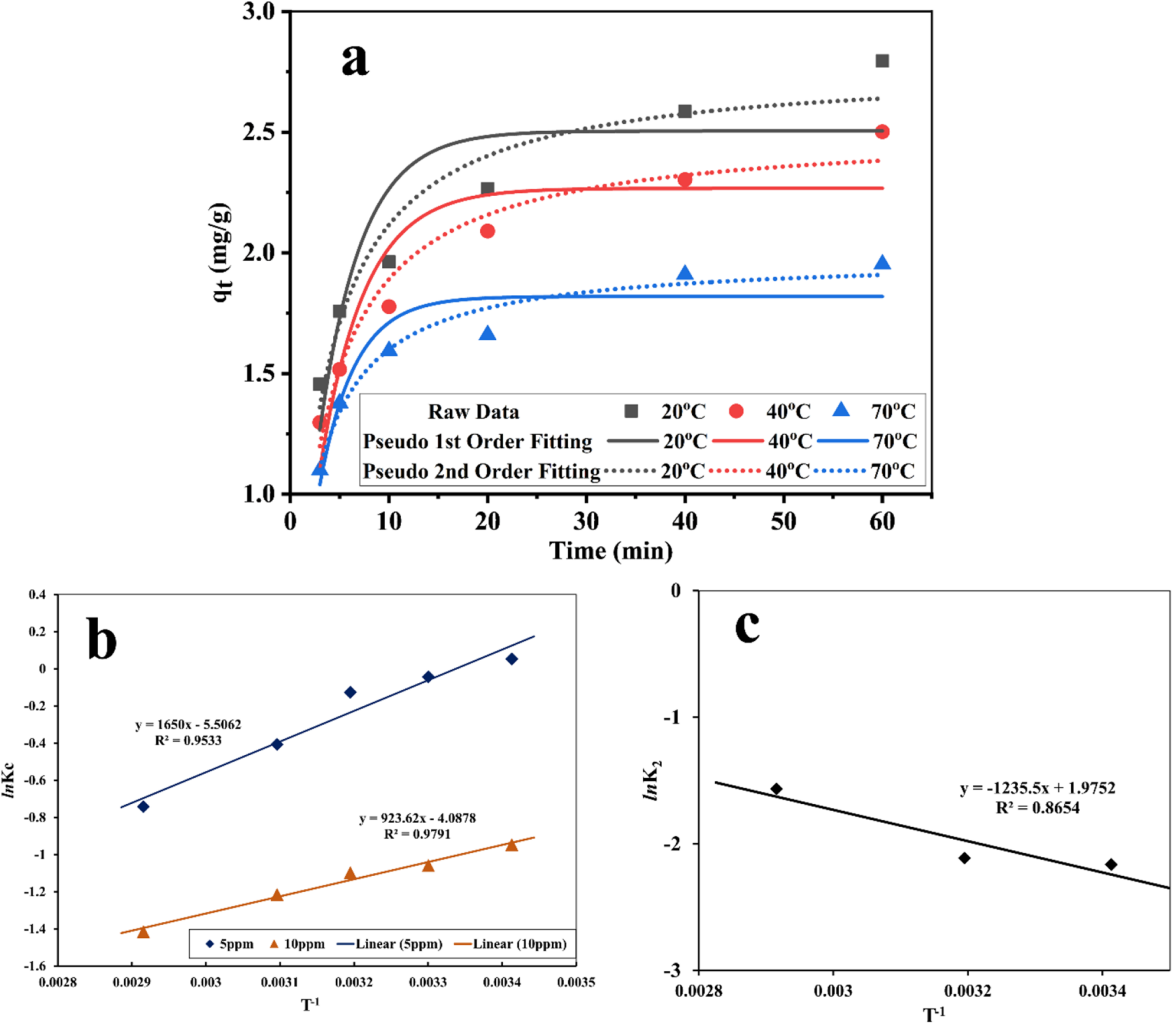
(**a**) Pseudo first-order and pseudo second-order kinetic models for degradation of 10 ppm MB in presence of sample S4 as catalyst and ZrO_2_ balls with 350 RPM in speed at different temperatures: 20 °C (black curve), 40 °C (red curve), and 70 °C (blue curve). (**b**) LNK Vs 1/T Plot for the experimental data for the evaluation of thermodynamic Parameters of the degradation of MB. (**c**) Activation energy for decomposition of MB by piezo catalyst based on Arrhenius equation.

Pseudo-second-order kinetic model assumes that the rate of degradation is second order^45,46^.

2 $$ \frac{dq}{{dt}} = {\text{k}}_{2} ({\text{q}}_{{\text{e}}} - {\text{q}}_{{\text{t}}} )^{2} $$

where k_2_ is the pseudo-second-order rate constant (Figs. 6, 7a). Tables 1 and 2 show the kinetic parameters at different shaker speeds and different temperatures (20, 40, and 70 °C) which were obtained from non-linear regression of the isotherm models. Regarding the shaking speed of 250 RPM at 303 K, the correlation coefficient is R^2^ = 0.79 for the first order kinetic model fitting which was low, while for the second order was about R^2^ = 0.94. It seems piezo degradation of MB was done through second order. By increasing the shaking speed to 350 RPM, R^2^ for the first and second order were close, R^2^ = 0.98 and 0.96 for the first and second order, respectively. R^2^ for the first and second order was still close by changing the mechanical force to the 250 W ultrasonic bath, 0.939 and 0.944 for the first and second order, respectively. It seems in lower shaker speed piezodegradation follows second order reaction while it follows first order in higher shaking speeds.

**Table 1:** Kinetic parameters for different mechanical forces in the decomposition of MB.

Kinetic models	Parameters	Different mechanical forces at 303 K		
		In water bath at 350 rpm	In sonication bath 320 W	In water bath at 250 rpm
Pseudo-first-order	*q*_*m*_ (mg g^−1^)	6.7	24.3	2.5
	*K*_1_ (min^−1^)	0.103	0.175	0.234
	*R*^2^	0.977	0.939	0.793
Pseudo-second-order	*q*_*e*_ (mg g^−1^)	8.1	4.9	2.8
	*K*_2_ (g mg^−1^ min^−1^)	0.013	0.044	0.115
	*R*^2^	0.959	0.944	0.940

**Table 2:** Kinetic parameters in the decomposition of MB for different temperatures.

Kinetic models	Parameters	Temperature (K)		
		293	313	343
Pseudo-first-order	*q*_*m*_ (mg g^−1^)	2.5	2.2	1.8
	*K*_1_ (min^−1^)	0.234	0.221	0.282
	*R*^2^	0.793	0.837	0.873
Pseudo-second-order	*q*_*e*_ (mg g^−1^)	2.7	2.5	1.9
	*K*_2_ (g mg^−1^ min^−1^)	0.115	0.121	0.209
	*R*^2^	0.940	0.961	0.967

Regarding the kinetic in different temperatures, R^2^ of 0.79 was obtained for first-order kinetic at 293 K while R^2^ of 0.94 was obtained for second-order kinetic at the same temperature. R^2^ was 0.84 and 0.87 at 303 and 313 K, respectively for first-order fitting. For second-order fitting R^2^ of 0.94 was achieved at 293 K. In the case of 303 K, R^2^ was about 0.96 by second-order fitting. Finally, R^2^ was about 0.97 at 313 K. As the results show, the reaction follows second order in higher reaction temperatures. In general, it seems the piezo degradation of MB by SbSI/Sb_2_S_3_ nanocomposites follows the second-order kinetic.

The thermodynamic parameters such as entropy (∆S°), Gibbs free energy (∆G°), and enthalpy (∆H^o^) for the degradation of MB by SbSI/Sb_2_S_3_ nanocomposites were calculated from the variation of Kc with temperature change of degradation and it can be determined from the following equations^47–49^:

3 $$ \Delta {\text{G}}^\circ = \Delta {\text{H}}^\circ - {\text{T}}\Delta {\text{S}}^\circ $$

4 $$ \Delta {\text{G}}^\circ = - {\text{RT}}\,{\text{ln}}\,{\text{K}}_{{\text{C}}} $$

5 $$ {\text{K}}_{{\text{C}}} = \frac{Ceq}{{CAe}} $$

6 $$ {\text{Ln}}\,{\text{K}}_{{\text{C}}} = \frac{\Delta S^\circ }{R} - \frac{\Delta H^\circ }{{RT}} $$

where ∆S° is the entropy change (kJ mol^−1^), ∆G° is the free energy change (kJ mol^−1^) and ∆H^o^ is the enthalpy change (kJ mol^−1^), Ceq = Concentration of dye at equilibrium (Reactant Conc. At equilibrium), Ae = concentration of dye depredated at equilibrium (Product Conc. At equilibrium) by SbSI/Sb_2_S_3_ nanocomposites at equilibrium. The thermodynamic parameters are tabulated in (Table 2). The negative value of ∆H^o^ indicates the exothermic degradation of MB by SbSI/Sb_2_S_3_ nanocomposites. ∆H^o^ decreased by increasing the MB concentration in the degradation process. ∆H^o^ is − 13.7 kJ mol^−1^ when MB concentration was 5 ppm and it changed to − 7.7 kJ mol^−1^ when MB concentration increased up to 10 ppm. ∆S° for 5 ppm of MB and 10 ppm of MB was − 0.0458 kJ mol^−1^ and − 0.0340 kJ mol^−1^, respectively. ∆G° for 5 and 10 ppm of MB at 293 K was − 0.305 kJ mol^−1^ and 2.279 kJ mol^−1^, respectively. By increasing the shaking temperature to 303 K, ∆G° increased to 0.153 and 2.619 kJ mol^−1^ for 5 ppm and 10 ppm of MB, respectively. ∆G° at 313 K, 323 K, and 343 K could be found in Fig. 7b and Table 3. According to the results, ∆G° increased by increasing the reaction temperatures. It means the piezo degradation reaction of MB is more favorable in lower temperatures. Figure 7c shows the activation energy based on the Arrhenius equation for the decomposition of MB by SbSI/Sb_2_S_3_ nanocomposites and it was 0.148 kJ mol^−1^.

**Table 3:** Thermodynamic parameters in the decomposition of MB for different temperatures.

Dye concentration (mg L^−1^)	∆H° (kJ mol^−1^)	∆S° (kJ mol^−1^)	∆G° (kJ mol^−1^)				
			293 K	303 K	313 K	323 K	343 K
5	− 13.7	− 0.0458	− 0.305	0.153	0.611	1.068	1.984
10	− 7.7	− 0.0340	2.279	2.619	2.959	3.298	3.978

## Conclusion

In summary, six types of SbSI/Sb_2_S_3_ nanocomposites synthesized via solvothermal and sonochemistry methods, whereas the SbSI/Sb_2_S_3_ nanocomposites prepared by sonochemistry (catalyst S_1_) in 2 h and SbSI/Sb_2_S_3_ nanocomposites prepared by solvothermal at 245 °C (catalyst S_4_) exhibit a higher piezocatalytic degradation performance. S_4_ decomposes 45.7% of MB when ultrasonic vibration with 250 W in power was used to stimulate piezo catalyst during 60 min of ultrasonication, while 89.1% of MB decomposes during 60 min by using ZrO_2_ balls as an alternative mechanical force to ultrasonic vibration. Changing shaking speed show dramatical effect on the degradation efficiency, so that degradation efficiency increased more than three times by increasing shaking speed from 150 to 350 RPM at 30 ± 5 °C. Temperature is another parameter that plays an important role in the degradation of MB by the SbSI/Sb_2_S_3_ nanocomposites. Degradation efficiency was increased by 42% by decreasing degradation temperature from 70 to 20 °C. Kinetic study shows that at a lower shaking speed of 250 RPM at 303 K, the correlation coefficients are R^2^ = 0.79 for the first-order kinetic model fitting which is low, while for the second order is about R^2^ = 0.94. It seems piezo degradation of MB was done through second order. By increasing the shaking speed to 350 RPM, R^2^ for the first and second-order are close, R^2^ = 0.98 and 0.96 for the first and second order, respectively. R^2^ for the first and second order is still close by changing the mechanical force to the 250 W ultrasonic bath, 0.939 and 0.944 for the first and second-order, respectively. It seems in lower shaker speed piezodegradation follows second order reaction while it follows first order in higher shaking speeds. As the results show, the reaction follows second order in higher reaction temperatures. In general, it seems the piezo degradation of MB by SbSI/Sb_2_S_3_ nanocomposites follows the second order kinetic. The negative value of ∆H^o^ indicates the degradation of MB by SbSI/Sb_2_S_3_ nanocomposites piezo catalyst is an exothermic process. According to the results, ∆G° increased by increasing the reaction temperatures. It means the piezo degradation reaction of MB is more favorable in lower temperatures.

## Supplementary Information

Supplementary Information.

## Data Availability

All data generated or analysed during this study are included in this published article [and its supplementary information files].

## References

[1] Frolova LA, Gutsev LG, Ramachandran BR, Dremova NN, Aldoshin SM, Troshin PA (2021). Exploring CsPbI3–FAI alloys: Introducing low-dimensional Cs2FAPb2I7 absorber for efficient and stable perovskite solar cells. Chem. Eng. J..

[2] Yu B-B, Chen Zh, Zhu Y, Wang Y, Han B, Chen G, Zhang X, Du Zh, He Zh (2021). Heterogeneous 2D/3D tin-halides perovskite solar cells with certified conversion efficiency breaking 14%. Adv. Mater..

[3] Tan D, Jiang Ch, Sun N, Huang J, Zhang Zh, Zhang Q, Bu J, Bi Sh, Guo Q, Song J (2021). Piezoelectricity in monolayer MXene for nanogenerators and piezotronics. Nano Energy.

[4] Li K, Sun E, Zhang Y, Song Zh, Qi X, Sun Y, Li J, Yang B, Liu J, Cao W (2021). High piezoelectricity of Eu3+-doped Pb(Mg_1/3_Nb_2/3_)O_3_–0.25PbTiO_3_ transparent ceramics. J. Mater. Chem. C.

[5] Cui Zh, Marcelle S, Zhao M, Wu J, Liu X, Si J, Wang Q (2022). Polyurethane/titania/polydopamine (TPU/TiO_2_/PDA) 3-D porous composite foam with outstanding oil/water separation performance and photocatalytic dye degradation. Adv. Compos. Hybrid Mater..

[6] Guo J, Chen Zh, El-Bahy ZM, Liu H, Abo-Dief HM, Abdul W, Abualnaja KhM, Alanazi AK, Zhang P, Huang M, Hu G, Zhu J (2022). Tunable negative dielectric properties of magnetic CoFe_2_O_4_/graphite-polypyrrole metacomposites. Adv. Compos. Hybrid Mater..

[7] Guo J, Li X, Chen Zh, Zhu J, Mai X, Wei R, Sun K, Liu H, Chen Y, Naik N, Guo Zh (2022). Magnetic NiFe_2_O_4_/polypyrrole nanocomposites with enhanced electromagnetic wave absorption. J. Mater. Sci. Technol..

[8] Moradi O, Madanpisheh MA, Moghaddas M (2021). Synthesis of GO/HEMA, GO/HEMA/TiO2, and GO/Fe3O4/HEMA as novel nanocomposites and their dye removal ability. Adv. Compos. Hybrid Mater..

[9] Xu Q, Gao X, Zhao S, Liu Y-N, Zhang D, Zhou K, Khanbareh H, Chen W, Zhang Y, Bowen Ch (2021). Construction of bio-piezoelectric platforms: From structures and synthesis to applications. Adv. Mater..

[10] Mohanta MK, Arora A, Sarkar AD (2021). Conflux of tunable Rashba effect and piezoelectricity in flexible magnesium monochalcogenide monolayers for next-generation spintronic devices. Nanoscale.

[11] Zhao Y, Gou G, Lu X, Hao Y (2021). Intrinsic auxeticity and negative piezoelectricity in two-dimensional group-IV dipnictide monolayers with in-plane anisotropy. J. Mater. Chem. C.

[12] Fu J, Xie A, Li T, Zuo R (2022). Ultrahigh piezoelectricity in (Ba, Ca)(Ti, Sn)O3 lead-free compounds with enormous domain wall contribution. Acta Mater..

[13] Tu Sh, Guo Y, Zhang Y, Hu Ch, Zhang T, Ma T, Huang H (2020). Piezocatalysis and piezo-photocatalysis: Catalysts classification and modification strategy, reaction mechanism, and practical application. Adv. Funct. Mater. Organoids Tissues.

[14] Lin P, Zhu L, Li D, Xu L, Pan C, Wang Zh (2018). Piezo-phototronic effect for enhanced flexible MoS_2_/WSe_2_ van der Waals photodiodes. Adv. Funct. Mater..

[15] Feng J, Sun J, Liu X, Zhu J, Xiong Y, Tian S (2019). Enhancement and mechanism of nano-BaTiO_3_ piezocatalytic degradation of tricyclazole by co-loading Pt and RuO2. Environ. Sci.: Nano.

[16] Sun J, Hua Q, Zhou R, Li D, Guo W, Li X, Hu G, Shan Ch, Meng Q, Dong L, Pan C, Wang ZhL (2019). Piezo-phototronic effect enhanced efficient flexible perovskite solar cells. ACS Nano.

[17] Amiri O, Abdalrahman A, Jangi G, Ahmed HA, Hussein SH, Joshaghani M, Mawlood RZ, Salavati-Niasar M (2022). Convert mechanical energy to chemical energy to effectively remove organic pollutants by using PTO catalyst. Sep. Purif. Technol..

[18] Choudhry I, Khalid HR, Lee H-K (2020). Flexible piezoelectric transducers for energy harvesting and sensing from human kinematics. ACS Appl. Electron. Mater..

[19] Aabid A, Raheman A, Ibrahim YE, Anjum A, Hrairi M, Parveez B, Parveen N, Zayan JM (2021). A systematic review of piezoelectric materials and energy harvesters for industrial applications. Sensors.

[20] Kim T, Cui Zh, Chang W-Y, Kim H, Zhu Y, Jiang X (2020). Flexible 1–3 composite ultrasound transducers with silver-nanowire-based stretchable electrodes. IEEE Trans. Ind. Electron..

[21] Ognibene G, Cristaldi DA, Fiorenza R, Blanco I, Cicala G, Scirè S, Fragalà ME (2016). Photoactivity of hierarchically nanostructured ZnO–PES fibre mats for water treatments. RSC Adv..

[22] Deak G, Dumitru FD, Moncea MA, Panait AM, Baraitaru AG, Olteanu MV, Boboc MG, Stanciu S (2019). Synthesis of ZnO nanoparticles for water treatment applications. Int. J. Conserv. Sci..

[23] Qian W, Zhao K, Zhang D, Bowen ChR, Wang Y, Yang Y (2019). Piezoelectric material-polymer composite porous foam for efficient dye degradation via the piezo-catalytic effect. ACS Appl. Mater. Interfaces.

[24] Raju TD, Veeralingam S, Badhulika S (2020). Polyvinylidene fluoride/ZnSnO_3_ nanocube/Co_3_O_4_ nanoparticle thermoplastic composites for ultrasound-assisted piezo-catalytic dye degradation. ACS Appl. Nano Mater..

[25] Xu X, Lin X, Yang F, Huang Sh, Cheng X (2020). Piezo-photocatalytic activity of Bi_0.5_Na_0.5_TiO_3_@TiO_2_ composite catalyst with heterojunction for degradation of organic dye molecule. J. Phys. Chem. C.

[26] Opoku F, Govender KK, Gertina C, van Sittert CE, Govender PP (2017). Recent progress in the development of semiconductor-based photocatalyst materials for applications in photocatalytic water splitting and degradation of pollutants. Adv. Sustain. Syst..

[27] Sarkar A, Adhikary A, Mandal A, Chakraborty T, Das D (2020). Zn-BTC MOF as an adsorbent for iodine uptake and organic dye degradation. Cryst. Growth Des..

[28] Cruz DRS, de Jesus GK, Santos CA, Silva WR, Wisniewski A, Cunha GC, Romão LPC (2021). Magnetic nanostructured material as heterogeneous catalyst for degradation of AB210 dye in tannery wastewater by electro-Fenton process. Chemosphere.

[29] Schneider J, Matsuoka M, Takeuchi M, Zhang J, Horiuchi Y, Anpo M, Bahneman DW (2014). Understanding TiO_2_ photocatalysis: Mechanisms and materials. Chem. Rev..

[30] Sakthivel S, Neppolian B, Shankar MV, Arabindoo B, Palanichamy M, Murugesan V (2003). Solar photocatalytic degradation of azo dye: Comparison of photocatalytic efficiency of ZnO and TiO_2_. Sol. Energy Mater. Sol. Cells.

[31] Xu T, Zhang L, Cheng H, Zhu Y (2011). Significantly enhanced photocatalytic performance of ZnO via graphene hybridization and the mechanism study. Appl. Catal. B.

[32] Maji TK, Bagchi D, Kar P, Karmakar D, Pal SK (2017). Enhanced charge separation through modulation of defect-state in wide band-gap semiconductor for potential photocatalysis application: Ultrafast spectroscopy and computational studies. J. Photochem. Photobiol. A.

[33] Altfeder I, Bianco E, Dorse DL (2018). Self-trapping and ordering of heavy holes in the wide band-gap semiconductor β-Ga_2_O_3_. Phys. Rev. B.

[34] Maeda K, Ishimaki K, Tokunaga Y, Lu D, Eguchi M (2016). Modification of wide-band-gap oxide semiconductors with cobalt hydroxide nanoclusters for visible-light water oxidation. Angew. Chem. Int Ed. Engl..

[35] Amiri O, Salar Kh, Othman P, Rasul T, Faiq D, Saadat M (2020). Purification of wastewater by the piezo-catalyst effect of PbTiO3 nanostructures under ultrasonic vibration. J. Hazard. Mater..

[36] Lin E, Kang Z, Wu J, Huang R, Qin N, Bao D (2021). BaTiO3 nanocubes/cuboids with selectively deposited Ag nanoparticles: Efficient piezocatalytic degradation and mechanism. Appl. Catal. B.

[37] Lei H, Wu M, Mo F, Ji S, Dong X, Jia Y, Wang F, Wu Z (2021). efficiently harvesting the ultrasonic vibration energy of two-dimensional graphitic carbon nitride for piezocatalytic degradation of dichlorophenols. Environ. Sci.: Nano.

[38] Manoharan S, Kesavan D, Pazhamalai P, Krishnamoorthy K, Kim S (2021). Ultrasound irradiation mediated preparation of antimony sulfoiodide (SbSI) nanorods as a high-capacity electrode for electrochemical supercapacitors. Mater. Chem. Front..

[39] Nowak M, Szperlich P, Bober Ł, Szala J, Moskal G, Stróż D (2008). Sonochemical preparation of SbSI gel. Ultrason. Sonochem..

[40] Pathak AK, Prasad MD, Batabyal SK (2019). Onedimensional SbSI crystals from Sb, S, and I mixtures in ethylene glycol for solar energy harvesting. Appl. Phys. A: Mater. Sci. Process..

[41] Dashairya L, Dasb De, Saha P (2021). Elucidating the role of graphene and porous carbon coating on nanostructured Sb_2_S_3_ for superior lithium and sodium storage. J. Alloys Compd..

[42] Li X, Bai J, Zhou B, Yuan X, Zhang X, Liu L (2018). High performance of 3D symmetric flowerlike Sb_2_S_3_ nanostructures in dye-sensitized solar cells. Chem. A Eur..

[43] Liu Z, Wu H, Zhuang J, Niu G, Zhang N, Rena W, Ye Z-G (2022). High Curie temperature bismuth-based piezo-/ferroelectric single crystals of complex perovskite structure: Recent progress and perspectives. CrystEngComm.

[44] Ning Zh, Jiang Y, Jian J, Guo J, Cheng J, Cheng H, Chen J (2020). Achieving both large piezoelectric constant and high Curie temperature in BiFeO_3_–PbTiO_3_–BaTiO_3_ solid solution. J. Eur. Ceram. Soc..

[45] Yang F, Zhu X, Wu J, Wang R, Ge T (2022). Kinetics and mechanism analysis of CO_2_ adsorption on LiX@ZIF-8 with core shell structure. Powder Technol..

[46] Wang H, Shen H, Shen C, Li Y, Ying Zh, Duan Y (2019). Kinetics and mechanism study of mercury adsorption by activated carbon in wet oxy-fuel conditions. Energy Fuels.

[47] Simonin J-P (2016). On the comparison of pseudo-first order and pseudo-second order rate laws in the modeling of adsorption kinetics. Chem. Eng. J..

[48] Xiao Y, Azaiez J, Hill JM (2018). Erroneous application of pseudo-second-order adsorption kinetics model: Ignored assumptions and spurious correlations. Ind. Eng. Chem. Res..

[49] Tran HN, You Sh-J, Chao H-P (2016). Thermodynamic parameters of cadmium adsorption onto orange peel calculated from various methods: A comparison study. J. Environ. Chem. Eng..

