# Supplementary material for: Kinetic and thermodynamic study in piezo degradation of methylene blue by SbSI/Sb2S3 nanocomposites stimulated by zirconium oxide balls
Source: Sci Rep. 2022 Sep 9;12:15242. doi: 10.1038/s41598-022-19552-3 (PMC9463189; doi:10.1038/s41598-022-19552-3)
Supplement: Supplementary file 1 — Supplementary Information. [file 41598_2022_19552_MOESM1_ESM.docx]

**Kinetic and thermodynamic study in piezo degradation of methylene blue by SbSI/Sb_2_S_3_ nanocomposites stimulated by Zirconium Oxide balls**

Omid Amiri ^a, b,*^, Karukh A. Babakr ^b^ , L. Jay Guo ^c^, Mohammad Ali Rashi ^b^ , Peshawa H. Mahmood ^a^

^a^ Faculty of Chemistry, Razi University, Kermanshah 6714414971, Iran

^b^ Chemistry Department, College of Science, University of Raparin, Rania, Kurdistan Region, Iraq

*^c^ Department of Electrical Engineering and Computer Science, University of Michigan, Ann Arbor, MI, USA*

* Corresponding author. Tel: +9647700581175

E-mail address: [o.amiri1@gmail.com](mailto:o.amiri1@gmail.com), [oamiri@uor.edu.krd](mailto:oamiri@uor.edu.krd)


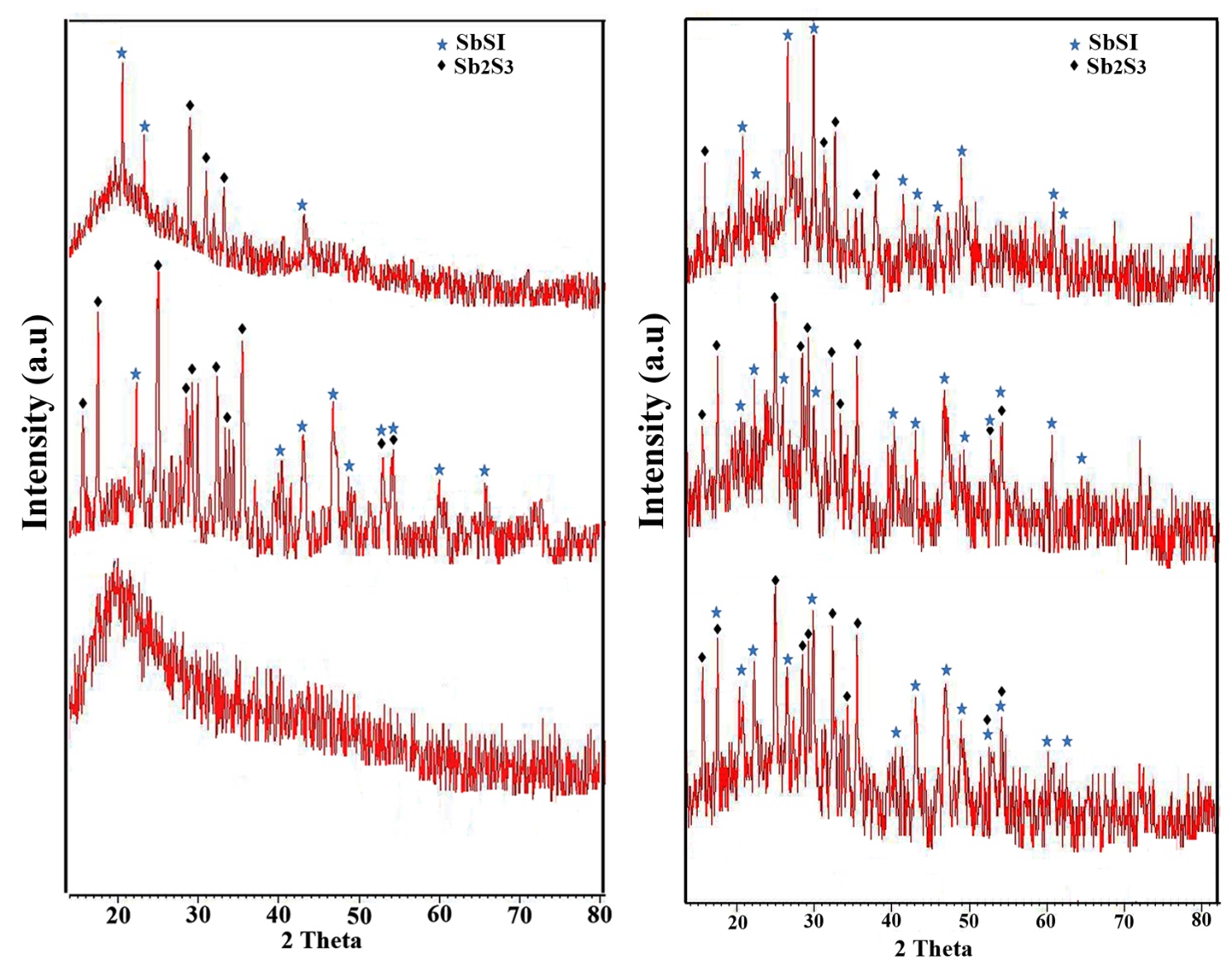


**Figure S1.** XRD patterns of prepared SbSI/Sb_2_S_3_ nanocomposite under different conditions.


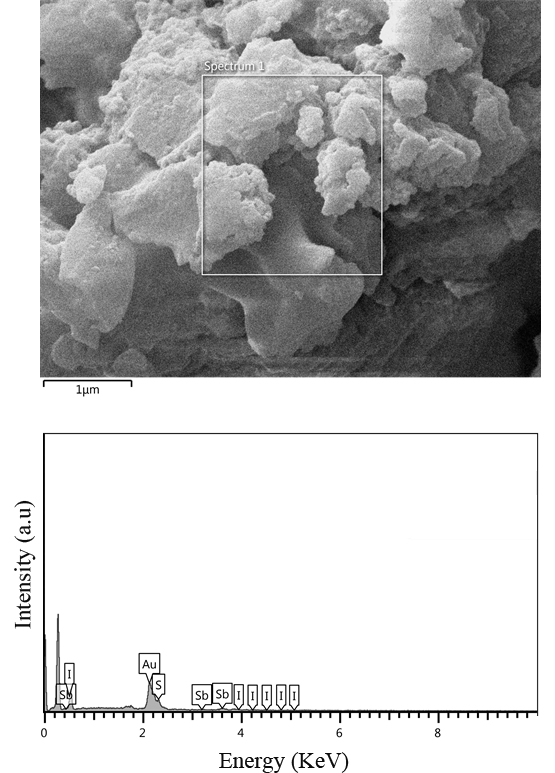


**Figure S2.** EDS image of sample S1.


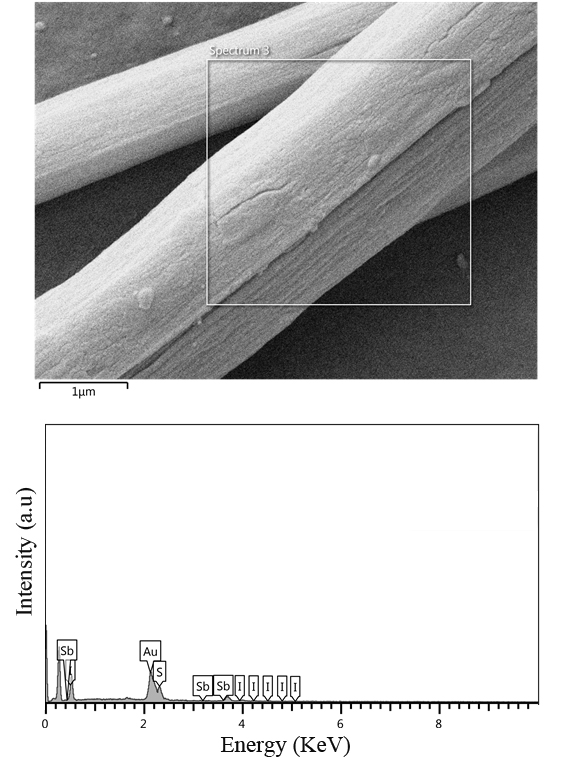


**Figure S3**. EDS images of sample S2.


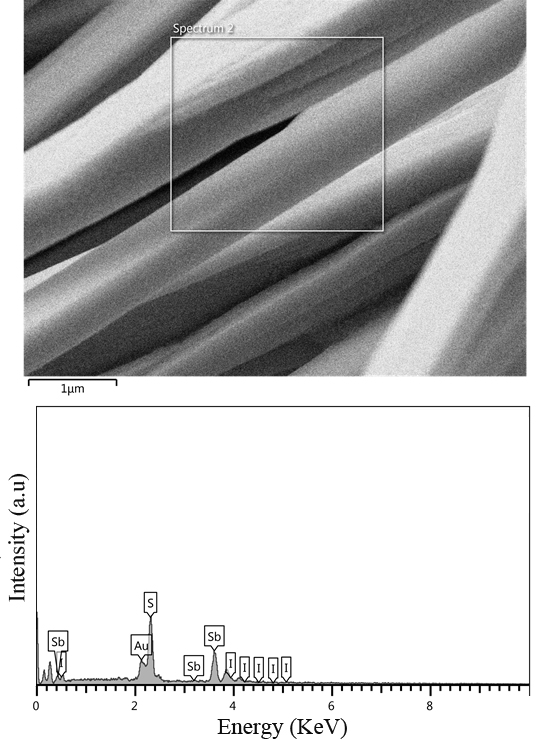


**Figure S4.** EDS image of sample S3.


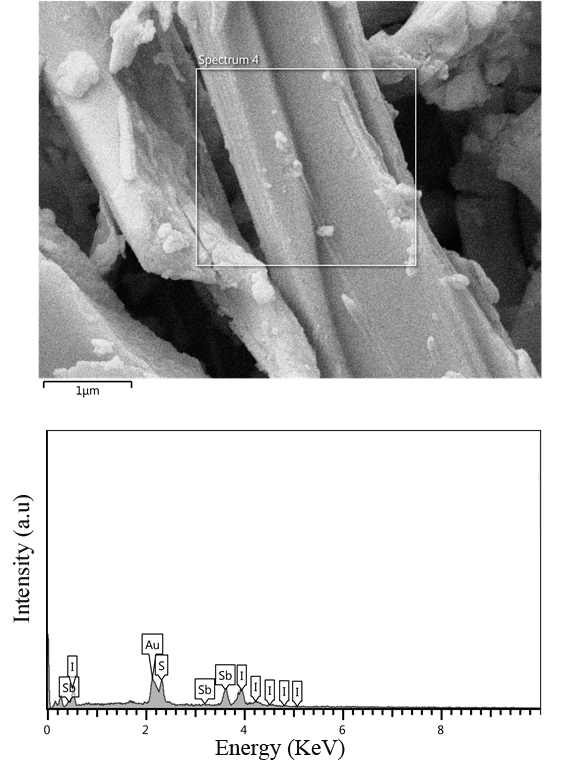


**Figure S5.** EDS image of sample S4.


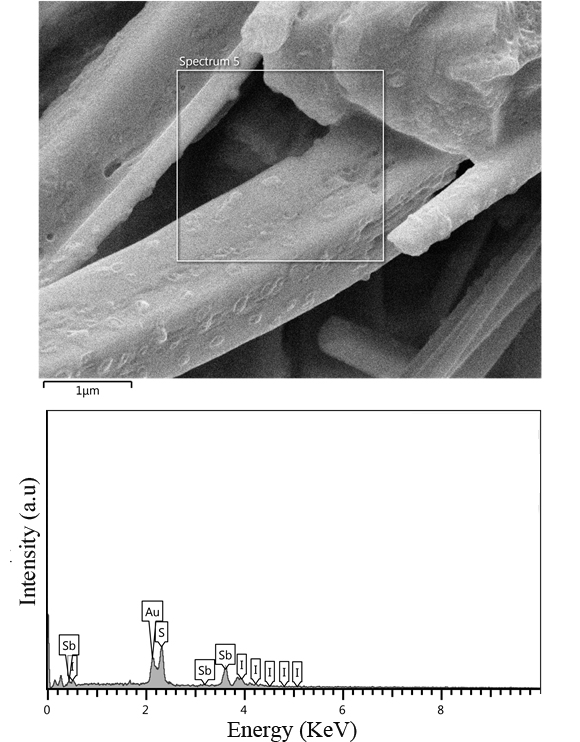


**Figure S6**. EDS image of sample S5.


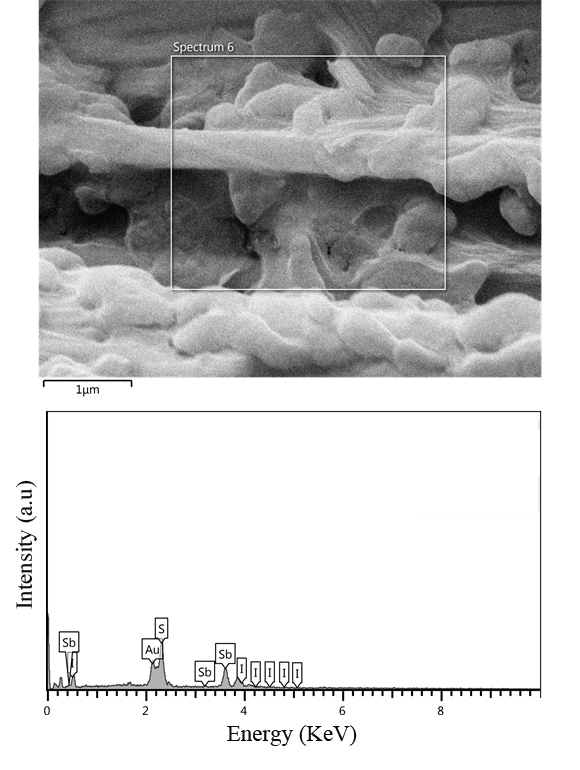


**Figure S7.** EDS image of sample S6.

We consider E_k_ as the initial energy transfer from the shaker to the MB solution when there are no balls in the dye solution [1-5].

E_k_=$\frac{1}{2}$ ρ ω^2^ (1)

Where ρ is the density of dye solution (fluid) and ω is angular velocity.

By adding n balls to the MB solution, E_k_ is constant and is divided between the balls and fluid. In this case, we have equation 2.

E_k_= $\frac{1}{2}$ ρ ω_2_^2^ + n ($\frac{1}{2}$ mω_2_^2^) (2)

Where ρ is density of fluid, ω_2_ is a new angular velocity after adding n balls, m is the average mass of each balls, and n is the number of balls added to the MB solution.

As equation 2 shows, ω will decrease by adding more balls to the MB solutions. We approve this by measuring ω in presence of 5, 10, and 15 balls. We measured the ball’s angular velocity by recording a slow-motion video and using a timer that could be found in a video in supporting information. Based on these results ω for 5, 10, and 15 balls were 250 RPM, 249.9 RPM, and 249.8 RPM.


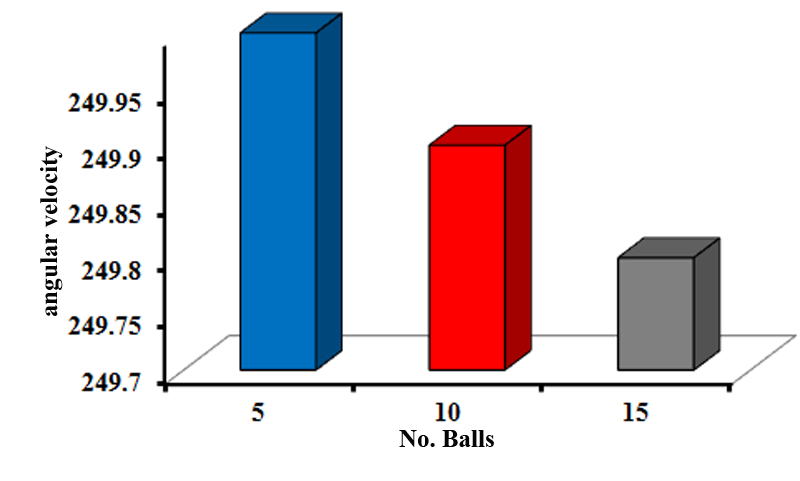


**Figure 8.** angular velocity while using 5, 10, and 15 balls.


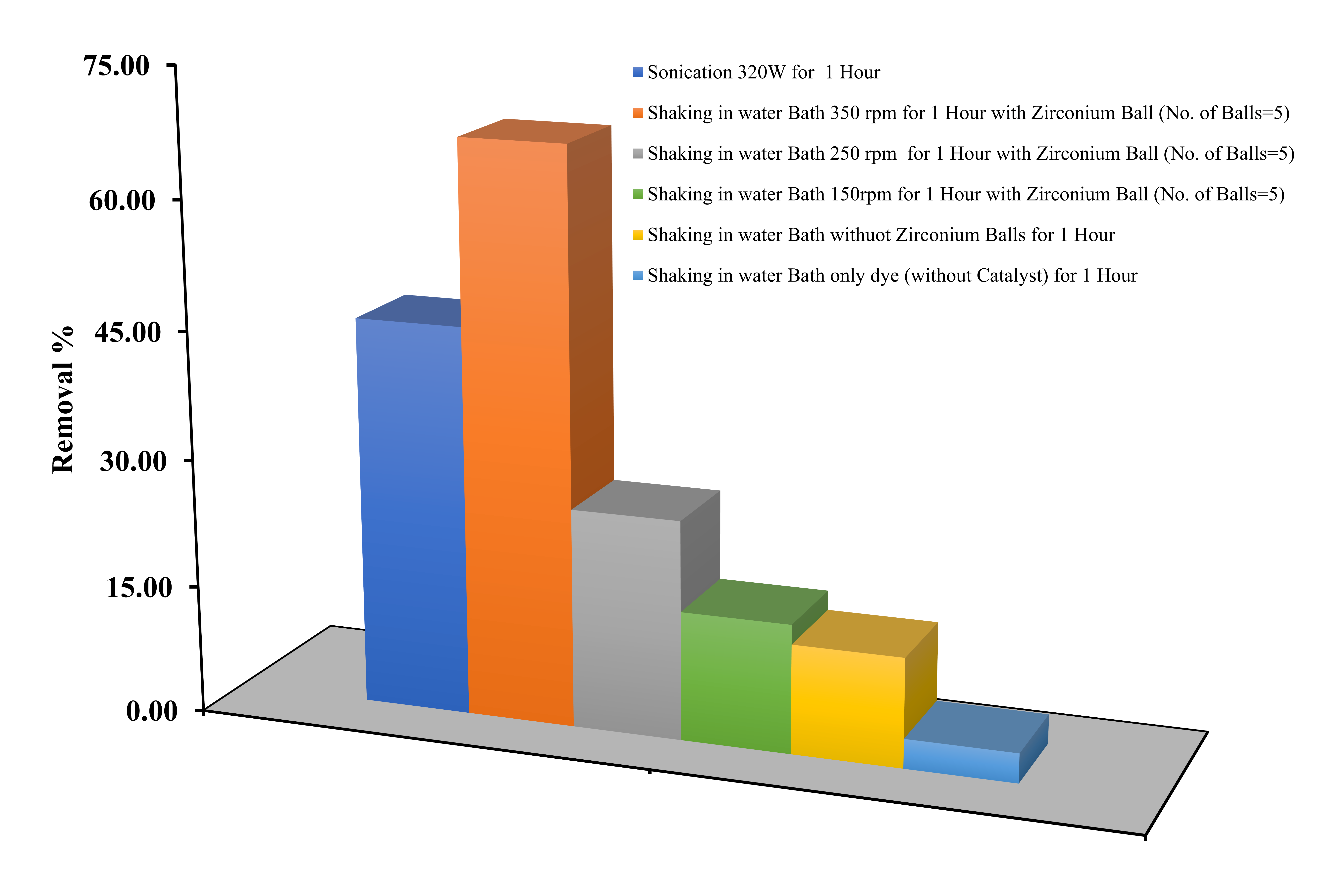


**Figure S9.** Effect of the type and amount of mechanical force on the degradation of MB.

**Table S1.** Experimental details for preparing samplesS1- S6.

| Sample Number | Preparation method | Sb precursor  (g) | S precursor  (g) | Iodine  (g) | Temperature  (℃) | Time  (h) | Ultrasonic power  (W) |
| --- | --- | --- | --- | --- | --- | --- | --- |
| S1 | Sonochemistry | 2 | 0.24 | 0.95 | 30 ± 5 | 2 | 250 |
| S2 | Sonochemistry | 2 | 0.24 | 0.95 | 30 ± 5 | 3 | 250 |
| S3 | Solvothermal | 2 | 0.24 | 0.95 | 180 | 6 | - |
| S4 | Solvothermal | 2 | 0.24 | 0.95 | 245 | 6 | - |
| S5 | Solvothermal | 2 | 0.24 | 0.95 | 180 | 4 | - |
| S6 | Solvothermal | 2 | 0.24 | 0.95 | 180 | 8 | - |

**References**

1. Y. Cui, J. Ravnik, M. Hriberšek, P. Steinmann, On Constitutive Models for the Momentum Transfer to Particles in Fluid-Dominated Two-Phase Flows, Advanced Structured Materials, 2018, 80, 1-25.

2. M. Rahmani, A. Hammouti, A. Wachs, Momentum balance and stresses in a suspension of spherical particles in a plane Couette flow, Physics of Fluids 2018, 30, 043301.

3. C. F. M. COIMBRA, R. H. RANGEL, General solution of the particle momentum equation in unsteady Stokes flows, Journal of Fluid Mechanics , 370 , 1998, 53 – 72.

4. W. M. Stacey, A Particle-, Momentum-, and Energy-Conserving Fluid Transport Theory for the Tokamak Plasma Edge, Fusion Science and Technology, 2019, 75, 251-263.

5. D. Gao, R. Fan, S. Subramaniam, D. Hoffman, Momentum Transfer Between Polydisperse Particles in Dense Granular Flow, 2006, 128, 62-68.
